# PCOS women show significantly higher homocysteine level, independent to glucose and E_2_ level

**Published:** 2016-08

**Authors:** Zahra Eskandari, Rajab-Ali Sadrkhanlou, Vahid Nejati, Gholamreza Tizro

**Affiliations:** 1 *Department of Biology, Faculty of Science, Urmia University, Urmia, Iran.*; 2 *Department of Comparative Histology and Embryology, Faculty of Veterinary Medicine, Urmia University, Urmia, Iran.*; 3 *Dr. Tizro Day Care and IVF Center, Urmia, Iran.*

**Keywords:** *Follicular fluid*, *IVF*, *Polycystic ovarian syndrome*, *Homocysteine*

## Abstract

**Background:**

It is reasonable to think that some biochemical characteristics of follicular fluid (FF) surrounding the oocyte may play a critical role in determining the quality of oocyte and the subsequent potential needed to achieve fertilization and embryo development.

**Objective:**

This study was carried out to evaluate the levels of FF homocysteine (Hcy) in IVF candidate polycystic ovary syndrome (PCOS) women and any relationships with FF glucose and estradiol (E_2_) levels.

**Materials and Methods:**

In this case control study which was performed in Dr. Tizro Day Care and IVF Center 70 infertile patients were enrolled in two groups: comprising 35 PCOS and 35 non PCOS women. Long protocol was performed for all patients. FF Hcy, glucose and E_2_ levels were analyzed at the time of oocyte retrieval.

**Results:**

It was observed that FF Hcy level was significantly higher in PCOS patients compared with non PCOSs (p<0.01). Observations demonstrated that in PCOS group, the Hcy level increased independent to E_2_, glucose levels, BMI and age, while the PCOS group showed significantly higher BMI compared with non-PCOS group (p=0.03). However, no significant differences were revealed between groups for FF glucose and E_2_ levels.

**Conclusion:**

Present data showed that although FF glucose and E_2_ levels were constant in PCOS and non PCOS patients, but the FF Hcy levels in PCOS were significantly increased (p=0.01).

## Introduction

Polycystic ovary syndrome (PCOS) is one of the most common female endocrine disorders affecting approximately 5-10% of women of reproductive age (12-45 years old) and is considered to be one of the leading causes of female subfertility (1). However, insulin resistance (IR) and elevated levels of homocysteine (Hcy) may be the major risk factors for the occurrence of atherosclerotic cardiovascular disease (CVD) in women with PCOS (2, 3). 

In order to improve the assisted fertilization techniques and to combine better results with reduced costs, some investigators have suggested oocyte retrieval without ovarian hyperstimulation. Immature retrieved oocytes are submitted to in vitro maturation followed by insemination, with a considerable reduction of labor and of operational and medication costs (4, 5). Thus, it is important to obtain detailed information about the oocyte environment, especially the composition and influence of Follicular fluid (FF) on the process of oocyte maturation, since the culture media could be improved by adding exogenous steroids oocytes. FF provides a very important microenvironment for oocyte development. FF is a product of both the transfer of blood plasma constituents that cross the blood follicular barrier and of the secretory activity of granulosa and theca cells (6). 

It is reasonable to think that some biochemical characteristics of FF surrounding the oocyte may play a critical role in determining the quality of oocyte and subsequent potential needed to achieve fertilization and embryo development. The analysis of FF components may also provide information on metabolic changes in blood serum, as the circulating biochemical milieu may be reflected in FF composition (7). FF is easily available as it is aspirated together with the oocyte at the time of oocyte pick-up (OPU). Homocysteine (Hcy) is an intermediated product formed by methionine breakdown and may undergo transsulfuration to cysteine and cystathionine.

Hcy is an essential amino acid required for the growth of cells and tissues. For humans, the only source of Hcy is methionine which is present in dietary proteins, and is mainly of animal origin. Folate and cobalamin (vitamin B_12_) are involved in Hcy remethylation, while pyridoxal 5'-phosphate (the active form of vitamin B_6_) is involved in Hcy transsulfuration (8). Many factors affect serum homocysteine levels including age, sex, nutrition, smoking, chronic inflammation, physical activity, and insulin (9-13). A significant inverse association between FF Hcy levels and oocyte and embryo quality is demonstrated in women undergoing assisted reproduction (14, 15). 

The composition of FF, to some extent, seems to reflect systemic Hcy metabolism, though high or low Hcy levels in the FF may well occur (16). Therefore ,various metabolic abnormalities in PCOS may also influence the quality of oocyte and embryo. It is possible that increased plasma Hcy levels could affect FF Hcy levels which, in turn, might affect the quality of oocyte and embryo in PCOS patients undergoing assisted reproduction.

This study was carried out to evaluate the FF Hcy levels in PCOS women IVF candidate and any relationships with FF glucose and estradiol (E_2_) levels.

## Materials and methods

**Study population**

This case control study enrolled 70 patients (aged 20-40 yrs) who received IVF treatment between September 2010 and May 2011 at Dr. Tizro Day Care and IVF Center, Urmia, Iran. The Research Ethics Committee of the Hospital approved the study and informed consent was obtained from all participants.

The study comprised 35 women among those attend the IVF center who were diagnosed with PCOS. PCOS was diagnosed if the ultrasound scan showed 10 or more cysts measuring 2-8 mm in diameter arranged peripherally around a dense core of stroma or scattered through an increased amount of stroma (17). A control group of 35 women among those who were attend the clinic for infertility management. The women in the control group and the oocyte donors had regular cycles and <10 follicles at the beginning of the cycle, with normal stomal volume.

Patients having any other major systemic illness including systemic inflammatory diseases, congenital adrenal hyperplasia, hyperprolactinaemia, and acromegaly were excluded from study. Patients whose FF was bloody during oocyte retrieval were excluded from study. All of the male partners had normal semen quality according to World Health Organization (WHO, 1999) criteria (18). PCOS patients with accompanying male factor infertility, endometriosis or tubal factor were excluded. All women were non-smokers and had been unable to be pregnant naturally for at least one year.

**Ovarian stimulation protocol**

The standard long protocol was used for ovarian stimulation. For pituitary suppression, each patient daily received 0.5 mg buserelin SC (Superfact, Aventis, Frankfurt, Germany) starting from day 21 of a spontaneous menstrual cycle (luteal phase). The administration of HMG hormone (Menogon, Ferring, Pharmaceuticals, Germany) was initiated on day 2 of the following menstrual cycle. 

The amount of injected hormone was based on age and body weight of patients. From the time of HMG administration, the dose of superfact was reduced to half amount given initially (0.25 mg/day). When two or more follicles reached a mean diameter of 18 mm, 10,000 IU of human chorionic gonadotropin (Pregnyl, Organon, Oss, the Netherlands) were administered. Oocyte retrieval was scheduled 36 hr after administration of hCG, using an ultrasound-guided transvaginal.

**Follicular fluid collection**

To avoid contamination from blood, flush medium or mixed FF during oocyte retrieval, only the FF from the first retrieved follicle from bilateral ovaries was collected. The presence or absence of blood contamination was graded by visual inspection, and samples that looked cloudy or blood stained were discarded: meticulous care was taken to include only uncontaminated samples. 

The collected FFs were processed by centrifugation at 3000 gr for 15 min at 4^o^C to eliminate cellular elements and subsequently frozen at -80^o^C until biochemical and hormonal analysis. The time elapsed between follicular aspiration and FF cryopreservation were not exceeded over 30 min. Then, FF Hcy, E_2_ and glucose levels were compared in both groups. Body mass index (BMI), was calculated as weight (kg) divided by height^2^ (m). 

**Biomarker measurements in follicular fluid**

Hcy, glucose and E_2_ levels were measured from FF at the time of oocyte retrieval. E_2_ was determined by an automated chemiluminescence technique (ELECYS 2010 HITACHI, Roche Diag. Germany, Diasorin kit). FF Hcy level was determined using an enzyme conversion immunoassay kit (Axis-Shield, Dundee, UK), and glucose was determined by enzymatic reaction technique [alkaline phosphatase kit (Pars Azmoon, Iran)].

**Statistical analysis**

Statistical analysis was performed using an SPSS package (version 16, Chicago, USA). Results reported as mean±SD. Correlation between variables was examined by Spearman’s correlation coefficient (r_s_) because the analyzed data were not normally distributed. However, p<0.05 was considered as statistically significant.

## Results

PCOS patients showed significantly higher concentration of Hcy (13.27±7.02) vs. control (9.29±2.68, p=0.01), BMI (28.13±5.25) vs. control (25.31±3.11, p=0.03), number of oocyte collected (16.17±9.56) vs. control (9.29±4.45, p=0.003) (Table I). The pregnancy rate in non PCOS patients were significantly higher than PCOS group (50% vs. 33% respectively) (Table I). We observed no marked difference in glucose level between patients with PCOS (70.25±12.77) vs. control (65.58±14.84) and E_2_ level between patients with PCOS (1291±334.61) vs. control (1217.5±307.73) (Table I). 

Tables II, III show relationship between FF Hcy concentration and selected parameters in PCOS patients and non PCOS group. No correlation was found between elevated BMI and Hcy level in two groups, and no correlation was found between Hcy level and age in two groups (Table II, III). The elevated level of Hcy in PCOS patients is independent of parameters such as E_2_ and glucose level, BMI and age. 

**Table I:** Comparison of the patient^’^s characteristics in PCOS women and non PCOS group

**Parameter **		**Mean±SD**	**p-value**
Follicular fluid Hcy (μmol/mL) *			
	Non PCOS	9.29 ± 2.68	0.01
	PCOS	13.27 ± 7.02	
BMI (kg/m^2^) *			
	Non PCOS	25.31 ±3.11	0.03
	PCOS	28.13 ± 5.25	
Age (year) *			
	Non PCOS	27.67 ± 5.03	0.99
	PCOS	30.21 ±5.41	
Follicular fluid E_2_ (pg/ml) *			
	Non PCOS	1217.5±307.73	0.80
	PCOS	1291 ±334.61	
Follicular fluid Glucose (mg/dl) *			
	Non PCOS	65.58 ±14.84	0.24
	PCOS	70.25 ± 12.77	
Fertilization rate (%) *			
	Non PCOS	85.44 ± 12.35	0.88
	PCOS	84.79 ± 12.78	
Number of oocyte collected (n) *			
	Non PCOS	9.29 ± 4.45	0.003
	PCOS	16.17 ± 9.56	
Pregnancy rate (%) **			
	Non PCOS	50%	
	PCOS	33%*	

* Data presented as mean ±SD.

** Data presented as n (%)

**Table II:** Correlation of follicular fluid Hcy concentration with selected reproductive parameters in PCOS patients

**Parameters**	**R**	**p-value**
Hcy and BMI	-0.07	0.73
Hcy and Age	0.31	0.12
Hcy and E_2_	-0.13	0.55
Hcy and Glucose	-0.13	0.51
Hcy and Number of oocyte collected	0.14	0.50

* Values are significant at p<0.05.

**Table III:** Correlation of follicular fluid Hcy concentration with selected reproductive parameters in on PCOS group

**Parameters**	**R**	**p-value**
Hcy and BMI	-0.17	0.42
Hcy and Age	0.14	0.49
Hcy and E_2_	0.24	0.24
Hcy and Glucose	-0.07	0.73
Hcy an Number of oocyte collected	0.03	0.89

* Values are significant at p<0.05.

## Discussion

This study was carried out to evaluate the levels of FF Hcy in IVF candidate PCOS women and any relationships with FF glucose and E_2_ levels. According to the findings of this study, FF Hcy level in PCOS group was higher than that in non-PCOS group and there was a significant difference between the two groups in this regard. Similarly, many studies have detected elevated plasma Hcy levels in women with PCOS (19-22). According to this study findings elevated level of Hcy in PCOS patients is independent of parameters such as E_2_, glucose level and age. So far, FF Hcy levels and the associations with FF estradiol have not been reported in PCOS patients undergoing assisted reproduction. This implies that Hcy elevation might be due to factors such as insulin resistance and relative hyperandrogenemia in PCOS patients. However, further studies are needed to investigate the effects of food or body composition and high genetic levels of Hcy in PCOS patients. 

The result of the present study showed that PCOS patients were more obese than the women in non PCOS group and BMI were significantly higher in PCOS group than in non PCOS (p<0.03). Our study indicated that the total mean number of oocyte retrieval from PCOS patients was significantly higher than that of the non PCOS group (p<0.003). Engman *et al* found similar results (23). It was observed that pregnancy rate in non PCOS patients was significantly higher than in PCOS patients (50% vs. 33% respectively). Therefore a conceptual agreement suggests that oocytes and embryos are of poor quality from patients with PCOS (24-29). 

Significant inverse association between FF Hcy levels and oocyte and embryo quality was demonstrated in women undergoing assisted reproduction (14, 15). The clinical and preclinical data suggest poor oocyte and embryo quality, and a lower fertilization rate in PCOS patients undergoing assisted reproduction (24, 29-32). FF Hcy levels may affect pregnancy outcome in PCOS patients undergoing assisted reproduction. However, several studies have previously found that fertilization rate was lower in PCOS patients whereas other studies reported that the fertilization rate was not affected (23, 24, 27, 28, 30-39).

Similarly, many studies showed no statistical significance in the level of FF estradiol between patients with PCOS and normally-ovulating infertile women in an IVF/ET program. Volpe *et al* compared the FF content of estrogen in a group of patients with polycystic ovary disease (PCOD) and normally-ovulating infertile women in an IVF/ET program (40). PCOD patients showed similar FF estradiol levels when compared with controls. Sadeghipour *et al*, Xia and Younglai and Orief *et al* found similar results which are in line with the results of our study (41-43). These data indicated a normal intrinsic potential of aromatase activity in ovaries from PCOS patients stimulated with gonadotropins and suggested that PCOS do not develop from inherent ovarian aromatase deficiency. 

## Conclusion

Our results indicate that although FF glucose and E_2_ levels were constant in PCOS and non PCOS patients, the FF Hcy levels in PCOS were significantly increased.

## note

***This article extracted from M.Sc. thesis. (Zahra Eskandari)***
